# Outcomes of interfacility critical care adult patient transport: a systematic review

**DOI:** 10.1186/cc3924

**Published:** 2005-12-01

**Authors:** Eddy Fan, Russell D MacDonald, Neill KJ Adhikari, Damon C Scales, Randy S Wax, Thomas E Stewart, Niall D Ferguson

**Affiliations:** 1 Fellow, Interdepartmental Division of Critical Care Medicine, University of Toronto, 399 Bathurst Street, F2-150, Toronto, Ontario, M5T 2S8, Canada; 2 Assistant Professor, Division of Emergency Medicine, Department of Medicine, Sunnybrook and Women's College Health Sciences Centre, University of Toronto, 2075 Bayview Avenue, Toronto, Ontario, M4N 3M5, Canada; 3 Medical Director, Research Program, Ontario Air Ambulance, 20 Carlson Court, Suite 400, Toronto, Ontario, M9W 7K6, Canada; 4 Instructor, Interdepartmental Division of Critical Care Medicine, and Department of Medicine, Division of Respirology, Sunnybrook and Women's College Health Sciences Centre, University of Toronto, 2075 Bayview Avenue, Rm B7-08, Toronto, Ontario, M4N 3M5, Canada; 5 Assistant Professor, Interdepartmental Division of Critical Care Medicine, and Department of Medicine, Division of Respirology, Mount Sinai Hospital, University of Toronto, 600 University Avenue, Suite 1818, Toronto, Ontario, M5G 1X5, Canada; 6 Associate Professor, Interdepartmental Division of Critical Care Medicine, and Department of Medicine, Division of Respirology, Mount Sinai Hospital and University Health Network, University of Toronto, 600 University Avenue, Suite 1818, Toronto, Ontario, M5G 1X5, Canada; 7 Assistant Professor, Interdepartmental Division of Critical Care Medicine, and Department of Medicine, Division of Respirology, University Health Network, University of Toronto, 399 Bathurst Street, Toronto, Ontario, M5T 2S8, Canada

## Abstract

**Introduction:**

We aimed to determine the adverse events and important prognostic factors associated with interfacility transport of intubated and mechanically ventilated adult patients.

**Methods:**

We performed a systematic review of MEDLINE, CENTRAL, EMBASE, CINAHL, HEALTHSTAR, and Web of Science (from inception until 10 January 2005) for all clinical studies describing the incidence and predictors of adverse events in intubated and mechanically ventilated adult patients undergoing interfacility transport. The bibliographies of selected articles were also examined.

**Results:**

Five studies (245 patients) met the inclusion criteria. All were case-series and two were prospective in design. Due to the paucity of studies and significant heterogeneity in study population, outcome events, and results, we synthesized data in a qualitative manner. Pre-transport severity of illness was reported in only one study. The most common indication for transport was a need for investigations and/or specialist care (three studies, 220 patients). Transport modalities included air (fixed or rotor wing; 66% of patients) and ground (31%) ambulance, and commercial aircraft (3%). Transport teams included a physician in three studies (220 patients). Death during transfer was rare (n = 1). No other adverse events or significant therapeutic interventions during transport were reported. One study reported a 19% (28/145) incidence of respiratory alkalosis on arrival and another study documented a 30% overall intensive care unit mortality, while no adverse events or outcomes were reported after arrival in the three other studies.

**Conclusion:**

Insufficient data exist to draw firm conclusions regarding the mortality, morbidity, or risk factors associated with the interfacility transport of intubated and mechanically ventilated adult patients. Further study is required to define the risks and benefits of interfacility transfer in this patient population. Such information is important for the planning and allocation of resources related to transporting critically ill adults.

## Introduction

Regionalization of care and the requirement for specialized resources result in the frequent need for interfacility transport of critically ill patients [1-3]. Although some of these patients may derive significant benefit from such a transfer, they may also be at considerable risk of transport-related morbidity and mortality [4-12]. The decision to initiate the interfacility transport of a critically ill patient must, therefore, be taken carefully. The impact of specific pre-transport and transport-related factors on morbidity and mortality are not well established, however, limiting the ability of clinicians to target particular patients where additional resources and care during transportation might be beneficial. For example, if high-risk patients could be reliably identified, they could undergo additional pre-transport resuscitation [13,14] and/or be accompanied by specially trained transport personnel with additional equipment in order to anticipate and reduce transport-associated risks [15-21].

Several professional societies have developed guidelines for the inter- and intrafacility transport of critically ill patients [22-25]; however, these guidelines focus primarily on general principles (for example, pre-transport stabilization, minimum transport equipment and medications) and the composition of the transport team, rather than risk stratification. Understanding which patients are most at risk while undergoing interfacility transport and the types of events that occur would be an important step in patient preparation and aligning resources (such as equipment and personnel) at the sending and receiving sites as well as during transportation. To this end, we conducted a systematic review of the literature to determine the adverse events associated with interfacility transport of mechanically ventilated adult patients, along with important pre-transport and transport-related prognostic factors.

## Methods

### Identification of trials

Our objective was to identify all relevant published clinical studies describing the incidence and predictors of adverse events in mechanically ventilated adults undergoing interfacility transport. We chose to study only intubated and mechanically ventilated patients in order to capture a well-defined group of critically ill patients with significant severity of illness.

*A priori*, we defined adverse events related to transportation as those that occurred during interfacility transport and up to 24 hours after arrival at the destination. A computerized MEDLINE (1966 to 10 January 2005) search was conducted using the following medical subject headings: 'transportation of patients', 'intubation, intratracheal', and 'respiration, artificial'. In addition, we searched the databases CENTRAL (first quarter 2005), EMBASE (1980 to 10 January 2005), CINAHL (1982 to 10 January 2005), HEALTHSTAR (1975 to 10 January 2005), and Web of Science (1945 to 10 January 2005) using the keywords: 'transport', 'ventilation', and 'intubation'. No language restrictions were applied. Bibliographies of all selected articles and review articles [26,27] on interfacility patient transport were examined for other relevant studies. This strategy was performed iteratively, until no new clinical trial citations were found on review of the reference lists of retrieved articles. Full details of the searches are available upon request.

### Study selection and data abstraction/analysis

The following selection criteria were used to identify published studies for inclusion in our analysis: clinical trial or cohort study or case-series (study design); all patients intubated and mechanically ventilated, and aged ≥ 18 years (study population); and interfacility transport (for example, from one health care facility to another health care facility). Interfacility transports between two sites of the same institution were included if the means of transportation involved air or ground ambulance.

Two reviewers (EF and RDM) independently applied the selection criteria and abstracted the data using standardized forms. The reviewers abstracted data on description of the cohort, methods, adverse events/outcomes, and transport-related interventions. We report descriptive data from individual trials as mean ± standard deviation, unless otherwise stated. Because of the paucity of studies and the heterogeneity in study populations and reported outcomes, we did not conduct a meta-analysis.

## Results

The combined computerized and bibliographic literature search yielded 599 potentially relevant studies, of which 24 articles were identified for more detailed review (Figure 1). Only five studies satisfied our inclusion criteria [28-32]. There was moderate initial agreement between reviewers for study inclusion (raw agreement = 0.80, chance-corrected agreement κ = 0.65 ± 0.16); all disagreements were resolved by consensus.

The five included studies (Tables 1 and 2) enrolled 245 critically ill patients (median 15; range 8 to 146) with a wide variety of diagnoses. All were case-series, two of which were prospective. The most common indication for interfacility transport was the need for investigations and/or specialist care not available at the referring institution (three studies, 220 patients) [28,29,31,32]. The results of the included studies are summarized in Table 3.

### Pre-transport characteristics

Only 1 study reported severity of illness (Sepsis-related Organ Failure Assessment (SOFA) [33] score of 10 ± 3) prior to transport [31]. Another study reported pre-transport arterial blood gas results from transported burn patients [28]. The other three studies provided little data on pre-transport status that would be useful in standardizing comparisons across patient groups.

### Transport characteristics

Modalities used for interfacility transport included air (fixed or rotor wing; 66% of patients) and ground (31%) ambulance, and commercial aircraft (3%). Transport teams included a physician in 3 studies (220 patients) [28,31,32]. In one study, 14 patients (21%) were transported in the prone position because of life-threatening hypoxemia [31]. Death during transport was rare (n = 1) [32]. No other adverse events or significant therapeutic interventions during transport were reported in any of the included studies.

### Post-transport characteristics

One study (not the same one that described pre-transport characteristics) reported severity of illness on arrival and outcomes following interfacility transport (mean Acute Physiology and Chronic Health Evaluation (APACHE) II [34] score of 17 ± 6; intensive care unit mortality 30%) [29]. The burn study reported the incidence of respiratory alkalosis on arrival (in 19%) and the survival rate to burn unit discharge (71%) [28]. The presence or absence of post-transport adverse events was not reported in the other three included studies.

## Discussion

The main finding of this systematic review is the paucity of studies examining adverse events and their associated risk factors in critically ill patients undergoing interfacility transport. The few published studies suggest that significant mortality or morbidity associated with interfacility transport of intubated adult patients is uncommon; however, there are significant limitations to the available data. First, the estimation of the incidence of adverse events is unreliable because all studies were case series (the majority of which were retrospective) that enrolled few transported patients. Second, associations between pre-transport variables and adverse outcomes could not be determined, both because pre-transport status was poorly documented, and because studies lacked standard definitions and methods for ascertaining adverse events. Finally, many studies only examined immediate or short-term adverse events (for example, during transport or on arrival), even though it is possible that later adverse events may also be associated with important transport-related factors (for example, barotrauma from exposure to high ventilatory pressures during transport may go unrecognized for several hours).

A number of factors may have contributed to the low morbidity of interfacility transport documented in this review. These include the possibility that some patients who were less severely ill were intubated and ventilated solely to facilitate safe transportation, thereby lowering the overall acuity of illness and likelihood of adverse events. The extent to which this practice occurred was not reported in any of the included studies. In addition, the composition of the transport teams may have had an influence. In three of the five included studies, the transport teams included a physician; in two of these the physician was a specialist (a burn surgeon and an intensivist). In addition, a nurse accompanied the patient in all four studies that reported transport team composition. Interfacility transport is increasingly becoming the jurisdiction of highly trained and specialized transport personnel [35-38], with at least one paediatric study demonstrating significantly decreased morbidity associated with the use of such teams [36]. Professional guidelines have suggested that transport of unstable critically ill adults should be accompanied by either a physician or a nurse, preferably with additional training and experience in transport medicine [22]. The results of our review may not have been the same if more data were available from transports without such individuals.

Although transport methods, distance, and time differ in intra-hospital transfers, the risks and types of adverse events for the patient may be similar to those undergoing inter-hospital transport [24,39,40]. Several studies of intra-hospital transfers of critically ill patients have reported transport-related complications [39-42]. In a recent study [42], 191 incidents related to intra-hospital transport were identified over a six year period. The majority of adverse events centered on patient-staff management issues and equipment problems that culminated in serious complications in 31% of reported incidents, including major physiological deterioration in 15% and death in 2% [42]. This relatively high rate of adverse events among reported incidents when intrafacility transport is subjected to close scrutiny further calls into question the validity of the results of our review. It seems likely that the potential for adverse events is significantly higher during air transport between two hospitals than on a trip to another department within the same hospital such as the radiology department. Alternatively, a possible explanation is that patients undergoing intra-hospital transports are sicker and/or the personnel associated with these transports are less experienced than inter-hospital transport teams.

Finally, we acknowledge that a limitation to the generalizability of our results is the restriction of our review to intubated and ventilated patients undergoing interfacility transport. In our attempt to identify and study a well-defined population of critically ill patients, we may have missed other patients at risk for adverse events during interfacility transport.

The lack of informative clinical studies evaluating the interfacility transport of critically ill patients is likely related to a variety of barriers in conducting research in this setting (Table 4). Clearly, deciding if patients will undergo interfacility transport by randomization is infeasible and unethical. Therefore, we believe that a multi-center, prospective observational cohort study is the methodology best suited to address the important questions raised by our review in this rapidly growing field of transit care medicine. In the design of such a study, attention would need to be paid to developing and validating consistent definitions for adverse events. In addition, extensive collaboration between the critical care and transport teams would be essential.

## Conclusion

Few data document the risks of interfacility transport. Until more robust risk assessment tools become available, common sense and physiological rationale will continue to guide the risk/benefit assessment of interfacility transport for individual patients. We believe that more research is required to document the prevalence of adverse events in critically ill patients during transport, and to elucidate the associated patient- and transport-related risk factors. Such research could form the basis of new strategies to optimize patient safety. In addition, better identification of patients at risk may allow for more efficient and effective alignment of transport-related resources, such as specialist retrieval teams and enhanced pre-transfer stabilization.

## Key messages

• 	Few data exist regarding the mortality, morbidity, and/or risk factors associated with these outcomes in intubated and mechanically ventilated adult patients undergoing interfacility transport.

• 	Further prospective study is required to define the risks and benefits of interfacility transfer in this patient population.

• 	Such information is important for the planning and allocation of resources related to transporting critically ill adults.

## Competing interests

The authors declare that they have no competing interests.

## Authors' contributions

EF, RDM, DCS, TES, and NDF conceived the study. All authors contributed to the study design and interpretation of the data. EF and RDM performed the literature search and abstracted the data. EF wrote the first draft of the manuscript, which was then revised for intellectually important content by all authors. All authors read and approved the final manuscript.

## Figures and Tables

**Table 1:** Characteristics of included studies

Study	*n*	Country	Study duration	Study design	Diagnoses
Barillo *et al*. (1997) [28]	146	USA	Nov 1987 to Sept 1994	Retrospective case series	Smoke inhalation
					Facial burn/injury
					Polytrauma
					Pneumonia
					Respiratory failure from other causes
Remond *et al*. (1998) [29]	10	France	July 1996 to Sept 1997	Prospective case series	Meningitis
					Gas gangrene
					Post-operative respiratory failure
					Carbon monoxide poisoning
					Liver transplantation
					Stroke
Orf *et al*. (2000) [30]	15	USA	Not reported	Prospective case series	Traumatic brain injury
Uusaro *et al*. (2002) [31]	66	Finland	1993 to 1999	Retrospective case series	Acute respiratory distress syndrome
					Respiratory failure from other causes
Veldman *et al*. (2004) [32]	8	Germany	Not reported	Retrospective case series	Pneumonia
					Guillain-Barre syndrome
					Intracranial tumor
					Intracranial hemorrhage
					Acute respiratory distress syndrome
					Anoxic brain injury
					Neurodegenerative disease

**Table 2:** Transport characteristics of included studies

Study	*n*	Transport provider	Indication for transport	Transport method	Transport distance/time^a^	Transport team
Barillo *et al*. (1997) [28]	146	Public	Need for investigation and/or specialist facilities	Air ambulance (fixed wing > 150 miles; helicopter < 150 miles)	Helicopter (100 miles); fixed wing (912 miles)	Burn surgeon, ICU RN, RT, and medical technician
Remond *et al*. (1998) [29]	10	Not reported	Not reported	Ground ambulance	117 minutes	Not reported
Orf *et al*. (2000) [30]	15	Private	Not reported	Helicopter	Not reported	RN and paramedic
Uusaro *et al*. (2002) [31]	66	Not reported	Need for investigation and/or specialist facilities	Ground ambulance	161 km (median); 161 minutes (median)	Intensivist, RN, and 2 paramedics
Veldman *et al*. (2004) [32]	8	Private	Repatriation; need for investigation and/or specialist facilities	Commercial airline	1,700-10,280 nautical miles; 250-1,315 minutes	MD and RN

^a^Mean transport distance and time are reported unless otherwise specified. ICU, intensive care unit; MD, medical doctor; RN, registered nurse; RT, respiratory therapist.

**Table 3:** Results of included studies

Study	*n*	Pre-transport characteristics	Transport characteristics or adverse events	Post-transport characteristics or adverse events
Barillo *et al*. (1997) [28]	146	Mean extent of burn injury 40% TBSA 99% had smoke inhalation injury	No in-flight instability, respiratory complications, or failure of ventilation reported	28 pts (19%) with respiratory alkalosis; 104 (71.2%) survived to burn unit discharge
Remond *et al*. (1998) [29]	10	90% sedated 50% with PaO_2_/FiO_2 _ratio < 200	No adverse events reported	No adverse events reported
Orf *et al*. (2000) [30]	15	80% manually ventilated	Median AVR 24 AVR ≥ 26 in 33.3% of pts AVR ≥ 30 in 26.7% of pts	Mean AVR was lower in mechanically ventilated pts (15 ± 3) versus manually ventilated pts (29 ± 12) (p = 0.01)
Uusaro *et al*. (2002) [31]	66	52 pts (79%) with ARDS PaO_2_/FiO_2 _ratio 64 ± 20 mmHg SOFA 10 ± 3	14 pts (21%) transported in prone position 59 pts (89%) required inotrope/pressor infusions	Overall ICU mortality 30% PaO_2_/FiO_2 _ratio 73 ± 27 mmHg APACHE II 17 ± 6
Veldman *et al*. (2004) [32]	8	All pts ventilator-dependent ≥ 11 days prior to transport OI ≤ 9.5 prior to transport	Unsuccessful CPR for in-flight cardiac arrest (n = 1)	Not reported

Data are mean ± standard deviation. APACHE II, Acute Physiology and Chronic Health Evaluation II; ARDS, acute respiratory distress syndrome; AVR, assisted ventilation rate; CPR, cardiopulmonary resuscitation; ICU, intensive care unit; SOFA, Sepsis-related Organ Failure Assessment; TBSA, total body surface area; OI, oxygenation index ((FiO_2 _× mean airway pressure/PaO_2_) × 100).

**Table 4:** Barriers to transport research and recommendations for future studies

Barriers/problems	Potential solutions/approaches
Lack of validated and feasible definitions for many transport-associated complications	Develop *a priori *definitions for transport-associated complications by expert consensus; validate these prospectively (for example, pilot study) or retrospectively (for example, chart review)
Difficulties consistently documenting pre-transport clinical status across multiple sending facilities	Standardization of pre-transport data collection by centralized form/checklist administered by transport personnel at time of patient retrieval and/or by telephone follow-up following arrival at receiving facility
Limited monitoring (for example, no blood tests or X-rays) and documentation during transport	Standardization of data collection (for example, physiological parameters) during transport by centralized form/checklist administered by transport personnel during transport
Under reporting of adverse events/errors due to a real or perceived culture of blame	Anonymous reporting and independent abstraction of documented adverse events/errors; achieve 'buy-in' from frontline staff through education and involvement in project development
Inability to identify an adequately matched, non-transported comparison group due to heterogeneous patient population transported to tertiary centers and inevitable selection bias of those chosen for transport to these centers	Use of a multi-center, prospective observational cohort study including a broad spectrum of referral institutions; study risk factors for transport-related adverse events
